# Case Report: Step-by-step procedures for total intracorporeal laparoscopic kidney autotransplantation in a patient with distal high-risk upper tract urothelial carcinoma

**DOI:** 10.3389/fonc.2023.1142819

**Published:** 2023-04-24

**Authors:** Guohao Wu, Haomin Li, Junqiang Li, Mubiao Chen, Lishan Xie, Huilan Luo, Zhihui Chen, Dongming Ye, Caiyong Lai

**Affiliations:** ^1^ Department of Urology, Sixth Affiliated Hospital of Jinan University, Dongguan, China; ^2^ Department of Urology, First Affiliated Hospital of Jinan University, Guangzhou, China; ^3^ Operating Room, Sixth Affiliated Hospital of Jinan University, Dongguan, China; ^4^ Institute of Kidney Surgery, First Affiliated Hospital of Jinan University, Guangzhou, China

**Keywords:** total intracorporeal laparoscopic surgery, kidney-sparing surgery, kidney autotransplantation, urothelial carcinoma, upper tract urothelial carcinoma

## Abstract

A 47-year-old man presented to the emergency department with right abdominal pain and a new onset of painless haematuria two weeks earlier. Urine cytology test results suggested urothelial carcinoma. Computed tomography urography (CTU) showed a filling defect in the lower right ureter with right hydronephrosis. Lymphadenopathy and any signs of metastatic disease were absent on CTU. Cystoscopy appeared normal. Creatinine level was also normal before surgery. After the treatment options were discussed, the patient chose to undergo 3D total intracorporeal laparoscopic kidney autotransplantation, bladder cuff excision, and segmental resection of the proximal two-thirds of the ureter based on the membrane anatomy concept. After more than one year of follow-up, the patient was in good health and showed no signs of haematuria. Surveillance cystoscopy and CTU examination showed no evidence of disease recurrence. Therefore, it is reasonable to assume that kidney-sparing surgery may be considered for carefully selected patients with high-grade upper tract urothelial carcinoma.

## Introduction

Upper tract urothelial carcinoma (UTUC) is a relatively rare disease with an estimated incidence of 2 in 100,000 per year (1). Radical nephroureterectomy (RNU) with bladder cuff excision has long been recognized as the gold standard for patients with localized UTUC. Kidney-sparing surgery (KSS) is the preferred approach for low-risk UTUC, which has tumour outcomes similar to those of RNU, irrespective of the status of the contralateral kidney. According to the most recent European Association of Urology (EAU) Guidelines on UTUC, kidney-sparing management may be considered for high-risk tumours limited to the distal ureter, and according to a systematic review and meta-analysis of the recent literature, segmental ureterectomy (SU) is suggested for high-grade ureteral UTUC in well-selected patients (2). Jeldres C (3), Bagrodia A (4) and Fang (2) also support this view.

We reported a carefully selected patient with a diagnosis of high-grade UTUC of the right distal ureter. After the treatment options were discussed, the patient chose to undergo complete intracorporeal laparoscopic kidney autotransplantation, bladder cuff excision, and segmental resection of the proximal two-thirds of the ureter, as well as postoperative bladder instillation immunotherapy with bacillus Calmette-Guerin (BCG). After more than one year of follow-up, the patient was in good health and showed no signs of haematuria. Surveillance cystoscopy and CTU examination showed no evidence of disease recurrence.

## Case report

**Figure 1:**
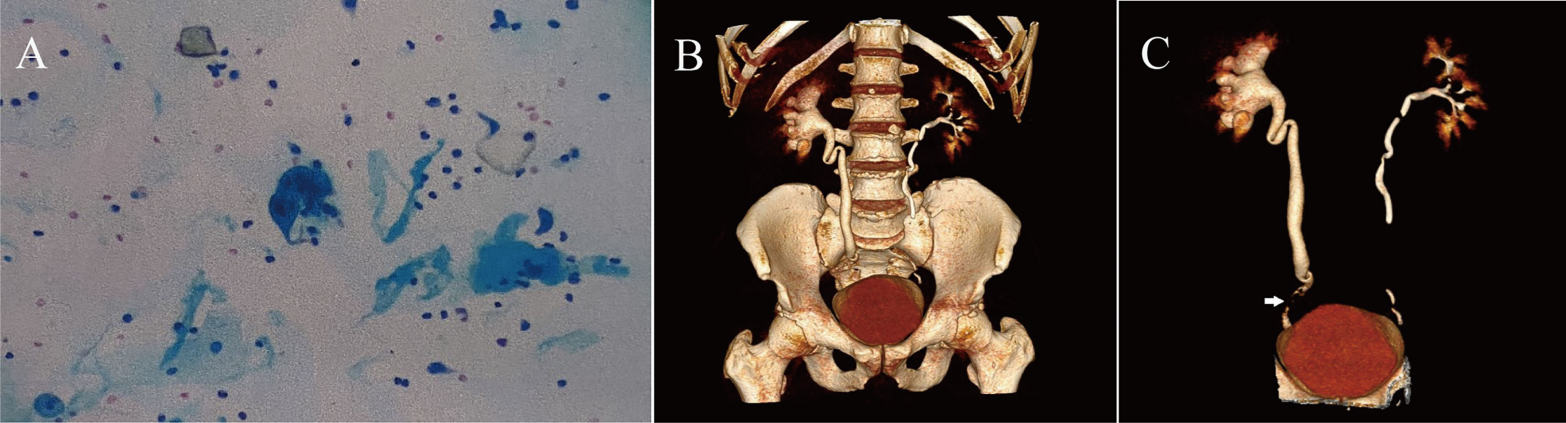
**(A)** The patient's preoperative urine cytologic testing results, suggesting urothelial carcinoma. **(B, C)** Preoperative CTU of the patient.

A 47-year-old man presented to the emergency department with a two-week history of pain in the right flank and new-onset painless haematuria. His medical history was notable for 30 pack-years of smoking. Physical examination revealed tenderness in the right flank. The results of the complete blood count and metabolic panel were normal. Creatinine and urea nitrogen were 0.87 mg/dl and 4.30 mmol/L, respectively. Urinalysis showed more than 15977 red cells per high-power field and more than 62 white cells per high-power field. The serum carcinoembryonic antigen result was 5.05 nanograms per millilitre (normal range, 0 to 5.0 nanograms per millilitre). A urine culture revealed Streptococcus agalactiae, and the results of urine cytologic testing suggested that the transitional cell carcinoma was arranged in a papillary structure with mild-moderate heterogeneity of cells, and urothelial carcinoma was considered (**Figure 1A**). CTU showed a filling defect in the lower right ureter with right hydronephrosis (**Figures 1B, C**, arrow). Preoperative examination of the bladder with a rigid cystoscope was unremarkable. After the treatment options were discussed, the patient chose to undergo 3D totally intracorporeal laparoscopic kidney autotransplantation, bladder cuff excision, and segmental resection of the proximal two-thirds of the ureter based on the concept of membrane dissection.

After general anaesthesia, a cystoscope was used to examine the bladder, and it showed normal mucosa and no tumour invasion at the bilateral ureteral outlet. The patient was placed in a 70° oblique position on the left side and treated with epirubicin 40 mg bladder irrigation chemotherapy for 30 minutes, and a 10 mm trocar and laparoscopic lens were placed through an incision at the right edge of the rectus abdominis muscle next to the umbilicus. A 12 mm and 5 mm trocar was placed under the costal margin in the midclavicular line and under the glabella, respectively, and a 5 mm trocar was placed at the intersection of the umbilical level and the right anterior axillary line and at the umbilicus and the right anterior superior iliac spine, respectively. The membrane was separated from the right kidney, right ureter, vena cava, and digestive system. The method used for membrane dissection in our study has been previously described in the literature (5).

**Figure 2:**
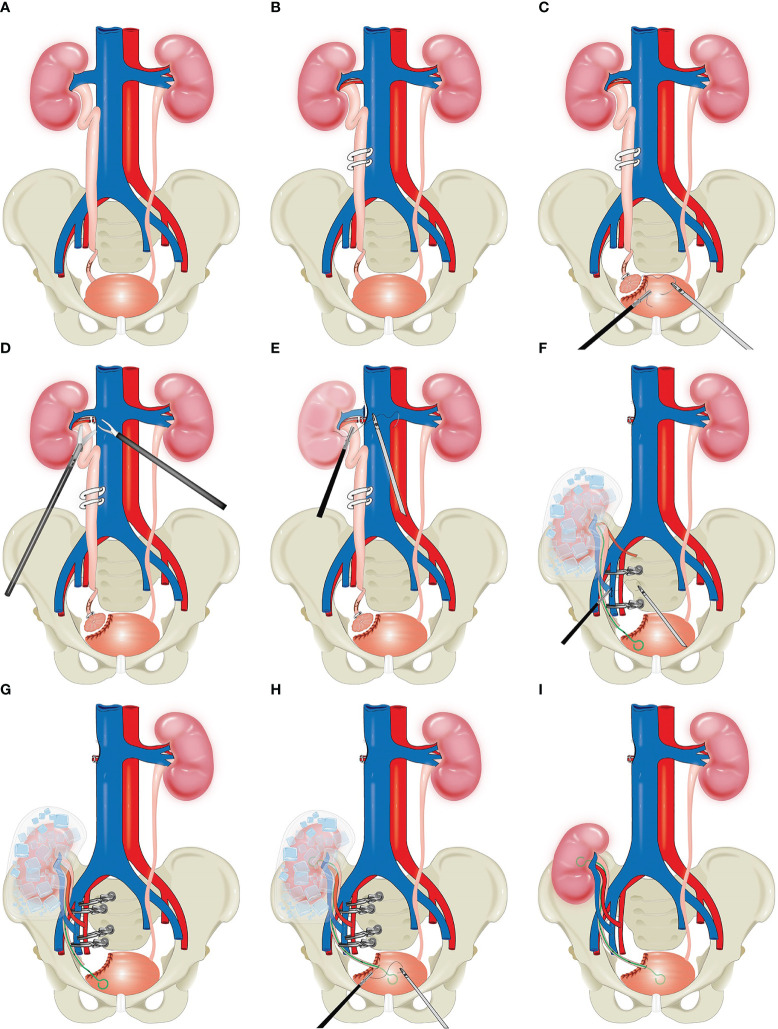
Schematic diagram of total intracorporeal laparoscopic kidney autotransplantation. **(A)** The tumor was located in the distal right ureter. **(B)** The right middle ureter was clamped using a hem-o-lock clip. **(C)** Bladder cuff excision. **(D, E)** The right renal artery and vein were fully clamped and disconnected, respectively, and the right vena cava was sutured. **(F, G)** Reconstruction of the vascular system after right kidney transplantation. **(H)** Reconstruction of the urinary tract system. **(I)** Kidney after transplantation.

First, the right Toldt's line was incised, and the digestive system was separated along the level between the posterior lobe of the right colonic mesentery and the anterior layer of Gorota's fascia as follows: superiorly to the base of the liver, inferiorly to the iliac vessels, and medially to the left side of the vena cava. Then, the right renal artery and vein were freed as well as the right ureter. The right middle ureter was clamped using a hem-o-lock clip (**Figures 2A, B**), the right ureter-bladder was cuffed and resected, and the cystotomy incision was sutured (**Figure 2C**). Then, the following lymph nodes were cleared sequentially: right hilar lymph nodes, right perirenal fat, paraventricular lymph nodes, right internal and closed foramen lymph nodes, and right external iliac lymph nodes. The right renal artery and vein were fully clamped and disconnected, respectively, and the right vena cava was sutured (**Figures 2D, E**). The right kidney and right ureter were quickly removed through an incision of approximately 7 cm in length in the right iliac fossa. We used a flexible ureteroscope in the intraoperative management of the kidney with the aim of confirming the presence of metastatic tumours in the renal pelvis and ureter. The right kidney and right renal vessels were trimmed under cold ischaemic hypothermia supplemented with small ice cubes, and a ureteral stent was placed in the right ureter. The right internal iliac vein was clamped at both ends by bulldog clips, and the right vein was reconstructed end-to-end with the right internal iliac vein (**Figure 2F**). Similarly, the right artery was reconstructed end-to-end with the right internal iliac artery (**Figure 2G**). Then, the right ureter was reconstructed end-to-end with the right posterior wall of the bladder (**Figure 2H**), and finally, the right internal iliac vein and arterial hobgoblin were released to restore blood flow to the right kidney, which was rich in vitality (**Figure 2I**). The right perirenal and pelvic drains were left in place, and each incision was sutured. The patient's operative time was 534 minutes, and the estimated intraoperative blood loss was 100 ml. The patient was discharged from the hospital on the 8th day after surgery.

Final pathological analysis revealed high-grade papillary ureteric carcinoma, with negative surgical margins, no invasion of the vasculature and nerves, and no metastasis in the lymph nodes (including right renal hilar, inferior vena cava parietal, right external iliac, right internal iliac, and closed hole). The pathological stage of the tumour was pT_2_N_0_M_x_. The patient was discharged from the hospital and instructed to take oral aspirin for anticoagulation therapy, and on the 5th day, painless haematuria appeared. Then, we performed "transurethral cystoscopy", and no significant bleeding was found in the bladder intraoperatively. We considered bleeding from the right uretero-vesical anastomosis due to postoperative oral aspirin. A regimen of 3 courses of gemcitabine 1.7 g in combination with cisplatin 120 mg chemotherapy was initiated on 23 January, 2022. The glomerular filtration rate (GFR) is a parameter representing kidney function. The GFR of the patient’s transplanted kidney was 48.6 ml/min at 4 months after surgery.

**Figure 3:**
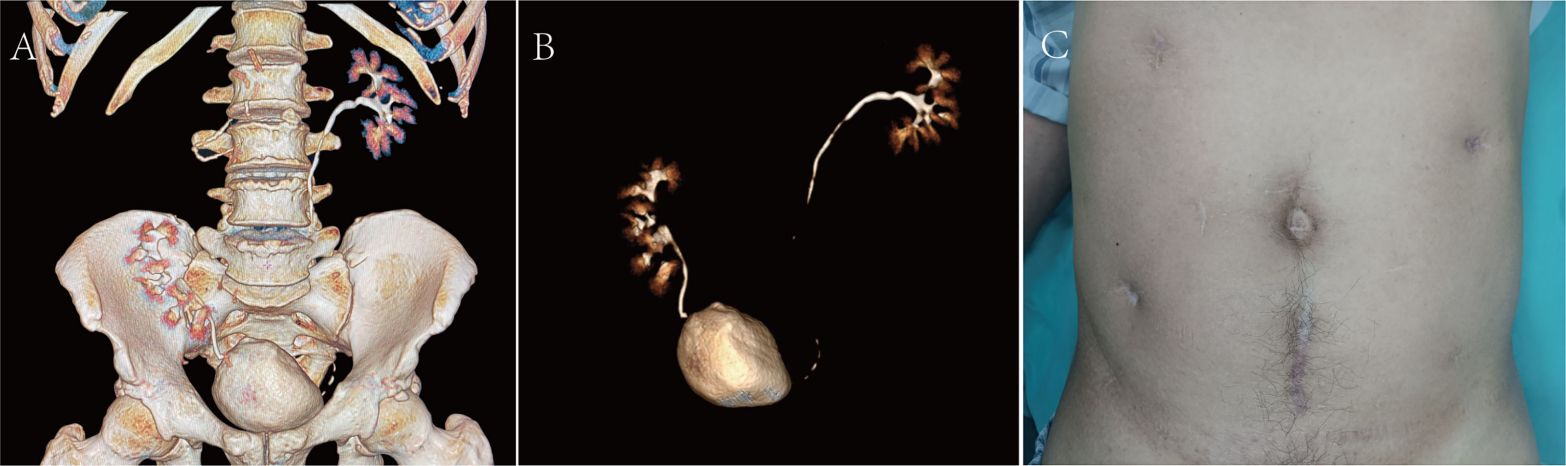
**(A, B)** CTU at the 1-year postoperative follow-up examination. **(C)** Postoperative cosmetic results.

**Figure 4:**
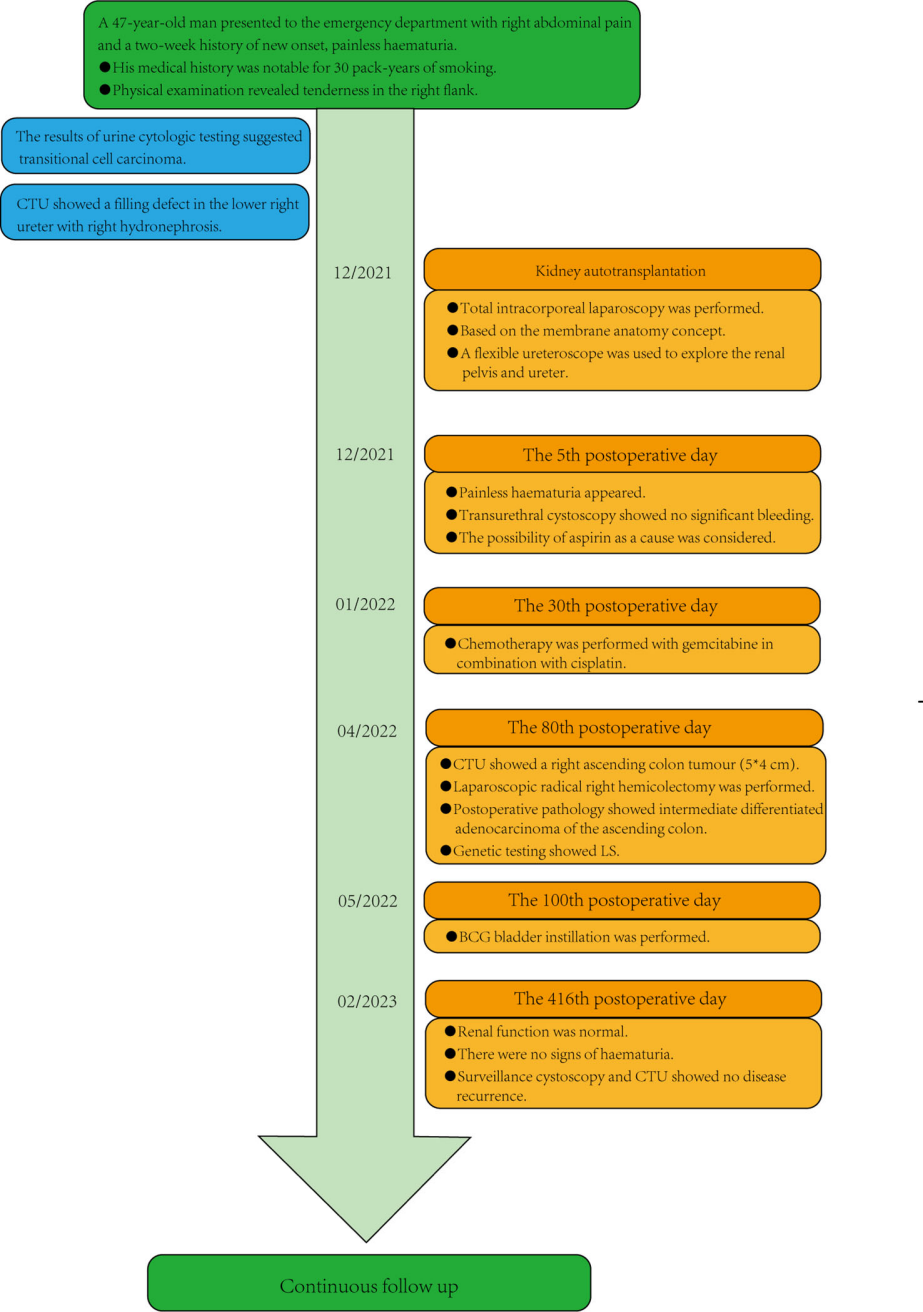
Patient timeline. CTU, computed tomography urography; LS, Lynch syndrome; BCG, Bacillus Calmette-Guerin.

On 18 April, 2022, the CTU examination was repeated and showed a right ascending colon tumour with a size of approximately 5*4 cm, so laparoscopic radical surgery was performed for a right hemicolectomy. The postoperative pathology suggested intermediately differentiated adenocarcinoma of the ascending colon, and the patient’s postoperative genetic test results suggested microsatellite instability (MSI) status of high microsatellite instability (MSI-H), and the immunohistochemistry (IHC) results showed mismatch repair deficiency (dMMR), indicating a diagnosis of Lynch syndrome (LS). Immunotherapy with 120 mg of BCG bladder instillation was started on 12 May, 2022. One year after the procedure, renal function was normal, with a creatinine level of 0.93 mg/dl. The patient was in good health and showed no sign of haematuria. Surveillance cystoscopy and CTU examination showed no evidence of disease recurrence (**Figures 3A, B**). The postoperative cosmetic results were satisfactory (**Figure 3C**), and the patient is still undergoing close follow-up (**Figure 4**).

## Patient perspective

I went to the hospital because of haematuria and right abdominal pain and found that I had urothelial carcinoma. The kidney autotransplantation surgery was very successful, and I am very satisfied. Now, I am back to my normal life. I hope that this technique can help more patients.

## Discussion

UTUC is a rare malignancy that accounts for 5% to 10% of all urothelial malignancies. Due to the insidious, multicentric, and aggressive nature of UTUC, the likelihood of recurrence is high (6). In addition, up to 10% of UTUC cases can be attributed to LS. LS is an autosomal dominant disorder caused by germline mutations in the MMR system that predispose patients to the early onset of the development of a variety of tumours (7). Therefore, individualized condition assessment for patient management is crucial, taking risk stratification and TNM tumour staging into consideration and adjusting the surgical strategy and technique according to the patient's condition and the risk of the tumour. According to recent EAU guidelines (1), low-risk UTUC manifests as the presence of a unifocal disease, a tumour size<2 cm, low-grade cytology, low-grade ureterorenoscopy (URS) biopsy, and no invasive aspect on computed tomography urography; high-risk UTUC is defined as the presence of at least one of the following: hydronephrosis, tumour size ≥2 cm, high-grade cytology, high-grade URS biopsy, multifocal disease, previous radical cystectomy for high-grade bladder cancer, and variant histology). KSS is recommended for patients with low-risk UTUC, a solitary kidney or impaired renal function and may be considered for patients with high-risk tumours limited to the distal ureter. To the best of our knowledge, the gold standard of treatment for high-risk UTUC is RNU. This radical approach is largely driven by the notion of "safety first" because staging this disease is difficult and potentially invasive (8). However, in a population-based study of 2299 patients, researchers found that nephrectomy and SU for invasive UTUC was effective in controlling cancer in patients with organ-confined (pT_1-2_) UTUC, nephroureterectomy (NU) or SU and that the cancer-specific mortality (CSM) rate in patients with pT_2_N_0_ stage was 86.2% (9). Furthermore, Hendriks N et al. (10) reported their largest cohort of oncological outcomes in patients with high-risk UTUC treated with KSS and concluded that not all high-risk UTUC cases need to be treated with RNU. In addition, a recent study by Abrate A (11) showed that patients treated with termino-terminal anastomosis or ureteric reimplantation for UTUC exhibited comparable 5-year overall survival (OS), cancer-specific survival (CSS), and recurrence-free survival (RFS), and in selected cases, termino-terminal anastomosis could be a safe solution if feasible.

In addition, similar results from a multi-institutional experience demonstrate that robotic SU and RNU are technically feasible in selected patients with nonmetastatic UTUC and can achieve favourable perioperative and oncologic outcomes (12). SU not only avoids surgically induced renal insufficiency and reduces the incidence of side effects associated with a solitary kidney, but it also preserves renal function, which may help improve the tolerance of adjuvant chemotherapy in patients with UTUC who are at high risk of recurrence and death (13). Therefore, it is reasonable to assume that KSS may be considered for patients with carefully selected high-grade UTUC, which was consistent with the findings of a recent study published by Kim TH (13). The precise preoperative staging of the patient is challenging in the management of UTUC due to practical and anatomical limitations (14, 15). The EAU guidelines consider endoscopic ablation as a potential treatment for low-risk UTUC with the main goal of total tumour resection or destruction. Although diagnostic URS biopsy is an important component in the diagnosis of UTUC, allowing for patient risk stratification, it can present a technical challenge. As invasive preoperative diagnostic modalities are associated with the risk of urinary bladder recurrence (UBR), URS biopsy is a significant risk factor for UBR in UTUC (16). Thus, we instead used a flexible ureteroscope to probe the renal pelvis and ureter of the hypothermic isolated kidney to further clarify the presence of tumour metastasis and implantation, which was a feasible approach.

There are several surgical treatment options to consider for UTUC of the distal ureter, such as distal ureterectomy with ureteroneocystostomy for low-risk tumours of the distal ureter that cannot be completely resected by endoscopy and high-risk tumours requiring renal function preservation for KSS, segmental resection of the distal ureter combined with a bladder flap, and total ureterectomy with ileal ureter replacement in selected cases. In our case, if the patient underwent distal ureterectomy with ureteroneocystostomy, the length of the ureter would be insufficient to achieve tension-free anastomosis; if the patient underwent distal ureterectomy with a bladder flap, the risk of tumour spillage would increase. Therefore, the surgical approach we chose was 3D total intracorporeal laparoscopic kidney autotransplantation with bladder cuff excision and segmental resection of the proximal two-thirds of the ureter based on the concept of membrane dissection, which was appropriate for this patient.

We have presented the carefully selected case of a patient who underwent KSS using 3D complete intracorporeal laparoscopy. The patient was found to have a right ascending colon tumour 4 months after surgery and underwent laparoscopic radical resection of right hemicolectomy for colon cancer. Postoperative pathology suggested intermediately differentiated adenocarcinoma of the ascending colon, and genetic testing suggested LS. After more than one year of follow-up, the patient was in good condition with no recurrence or metastasis. To our knowledge, this approach based on the concept of membrane dissection is the first application of 3D complete intracorporeal laparoscopic kidney autotransplantation combined with bladder cuff excision and segmental resection for distal ureteral tumours. The advantage of this procedure is that it allows for a relatively clear surgical field to be maintained, as we previously reported (5), as well as better postoperative cosmetic results.

To prevent recurrence and metastasis of UTUC, this patient underwent postoperative gemcitabine combined with cisplatin chemotherapy and immunotherapy with BCG bladder instillation. As a single therapy for bladder cancer, Gemcitabine has been extensively studied. Gemcitabine combined with cisplatin has similar efficacy to the M-VAC (cisplatin, methotrexate, doxorubicin, and vinblastine) regimen but is less toxic. Given that both cisplatin and gemcitabine, albeit *via* different mechanisms, cause DNA damage, one possible strategy is to interfere with DNA repair processes (17). The total response rate for the two-drug combination of gemcitabine and cisplatin varied from 41% to 71% (18–20). Because the standard treatment for metastatic urothelial carcinoma is the combination of gemcitabine and cisplatin (GC), it remains the first line of treatment for medically eligible patients (21). A recent randomized controlled study (22) found that starting gemcitabine-platinum combination chemotherapy within 90 days following NU significantly improved disease-free survival in patients with locally advanced UTUC. Data from a recent prospective multicentre phase II clinical trial support the use of neoadjuvant chemotherapy (NAC) as a standard of care for high-risk UTUC (23).

Although we did not perform RNU, we performed BCG bladder perfusion immunotherapy on our patients, considering that the bladder recurrence rate after RNU in UTUC is 22-47% (24, 25) and that BCG is the most effective therapy for high-risk non-muscle-invasive bladder cancer. The control mechanism of BCG in uroepithelial carcinoma involves a complex and multifaceted immune response. On the one hand, BCG enhances the effector function of tumour-specific CD4+ T cells by enhancing activation/differentiation and attenuating checkpoint blockade (26), aiming to play an auxiliary role in supplementing the original adaptive antitumour response, and on the other hand, BCG enhances the innate immune response by inducing epigenetic changes in monocytes and natural killer cells (27). The results of a randomized controlled study showed that for non-muscle-invasive bladder cancer, the complete response rate of standard-dose BCG was 85% (28). In addition, a recent prospective phase II study by Herr H for high-risk non-muscle-invasive bladder cancer showed an RFS rate of 85% at 2 years following complete remission with BCG bladder perfusion immunotherapy (29).

On the LS tumour spectrum, UTUC is the third most common malignancy. The most common form of familial colorectal cancer is LS, which is associated with changes in four DNA MMR genes: MSH2 plus EpCAM, MLH1, MSH6, and PMS2 (30). The optimal surveillance protocol for LS is to recommend that patients undergo a colonoscopy every 1-2 years, and the main method of surgical management is segmental or extensive colectomy. Recent reports in the literature (31) have indicated that the OS rates of colorectal cancer patients with LS are approximately 90% at 5 years, 80% at 10 years, and 70% at 15 years, which appear to be better than the survival outcomes of UTUC. LS is diagnosed mainly by genetic testing; however, the demographics that suggest the need for special attention or genetic testing remain unclear. Reports in the literature primarily discuss populations of UTUC patients younger than 65 years of age, with a personal history of LS-associated cancer, with one first-degree relative (FDR) younger than 50 years of age with LS-associated cancer, or with two FDRs with LS-associated cancer regardless of age (7).

Through careful evaluation and careful selection of patients with high-grade UTUC distal to the ureter, kidney autotransplantation *via* segmental resection combined with bladder cuff excision is an incredibly promising option, according to an analysis of this case's characteristics and a review of the literature. First, segmental resection combined with bladder cuff excision for autologous kidney autotransplantation allows complete removal of the tumour; second, the greatest advantage of kidney autotransplantation is the preservation of renal function, which avoids the dilemma of choosing between renal dialysis or kidney transplantation and improves the tolerance of UTUC to adjuvant chemotherapy after surgery; finally, total intracorporeal laparoscopic kidney autotransplantation necessitates a short postoperative hospital stay, allows rapid recovery, and provides more satisfactory cosmetic results. From our point of view, although the technique of KSS is feasible, given the characteristics of UTUC tumours, it is important to develop a proper surveillance strategy to better evaluate patients and detect recurrent tumours. According to the latest EAU guidelines (1), close follow-up is recommended at 3 and 6 months and then annually for patients undergoing KSS, including cystoscopy, urine cytology, CT urography and chest CT; in addition, URS and *in situ* urine cytology were performed at 3 and 6 months. With regard to the patient follow-up regimen, patient follow-up is mandatory, and the latest EAU guidelines for close follow-up must be followed. The patient described herein is still undergoing close follow-up.

A few limitations of this study are worth considering. First, this was a pilot study; therefore, prospective randomized controlled studies at multiple centres with larger samples and longer follow-up periods are needed in the future. Additionally, this procedure requires relatively advanced laparoscopic skills and multidisciplinary cooperation and is not suitable for junior physicians.

In summary, we present the case of a patient with high-grade UTUC in the distal ureter treated by a KSS approach. The patient is still undergoing follow-up, and there is no sign of recurrence or metastasis. A longer period of observation is needed to determine this patient’s prognosis. Therefore, individualized treatment strategies and surgical conversion have benefited some suitable patients with high-grade UTUC of the distal ureter.

## Data availability statement

The original contributions presented in the study are included in the article/supplementary material, further inquiries can be directed to the corresponding author.

## Ethics statement

This study was approved by the Ethics Committee of the Sixth Hospital of Jinan University. Written informed consent was obtained from the participant/patient(s) for the publication of this case report.

## Author contributions

GW: manuscript writing, acquisition of data, analysis and interpretation of the data. HuL: manuscript writing, supervision. JL, LX and MC: assistance with data collection. HuL, ZC, and DY: manuscript review. CL: project development, management, operations, supervision. All authors contributed to the article and approved the submitted version.
